# SEOM-GG clinical guidelines for the management of germ-cell testicular cancer (2023)

**DOI:** 10.1007/s12094-024-03532-2

**Published:** 2024-07-03

**Authors:** José Angel Arranz Arija, Xavier García del Muro, Raquel Luque Caro, María José Méndez-Vidal, Begoña Pérez-Valderrama, Jorge Aparicio, Miguel Ángel Climent Durán, Cristina Caballero Díaz, Ignacio Durán, Enrique González-Billalabeitia

**Affiliations:** 1 Hospital General Universitario Gregorio Marañón, Madrid, Spain; 2 Hospital Duran I Reynals, Institut Català D’Oncologia L’Hospitalet (ICO), Barcelona, Spain; 3 Hospital Universitario Virgen de Las Nieves, Instituto de Investigación Biosanitaria Ibs, Granada, Spain; 4 Hospital Universitario Reina Sofía, Córdoba, Spain; 5 Hospital Universitario Virgen del Rocío, Seville, Spain; 6 Hospital Universitario I Politècnic La Fe, Valencia, Spain; 7 Instituto Valenciano de Oncología, Valencia, Spain; 8 Consorcio Hospital General Universitario de Valencia, Valencia, Spain; 9 Hospital Universitario Marqués de Valdecilla, Santander, Spain; 10 Hospital Universitario 12 de Octubre, IMAS 12, UCAM, Madrid, Spain

**Keywords:** Testicular cancer, Germ cell tumor, Chemotherapy, Surgery, Long-term survivors, Multidisciplinary teams

## Abstract

Testicular germ cell tumors are the most common tumors in adolescent and young men. They are curable malignancies that should be treated with curative intent, minimizing acute and long-term side effects. Inguinal orchiectomy is the main diagnostic procedure, and is also curative for most localized tumors, while patients with unfavorable risk factors for recurrence, or those who are unable or unwilling to undergo close follow-up, may require adjuvant treatment. Patients with persistent markers after orchiectomy or advanced disease at diagnosis should be staged and classified according to the IGCCCG prognostic classification. BEP is the most recommended chemotherapy, but other schedules such as EP or VIP may be used to avoid bleomycin in some patients. Efforts should be made to avoid unnecessary delays and dose reductions wherever possible. Insufficient marker decline after each cycle is associated with poor prognosis. Management of residual masses after chemotherapy differs between patients with seminoma and non-seminoma tumors. Patients at high risk of relapse, those with refractory tumors, or those who relapse after chemotherapy should be managed by multidisciplinary teams in experienced centers. Salvage treatment for these patients includes conventional-dose chemotherapy (TIP) and/or high-dose chemotherapy, although the best regimen and strategy for each subgroup of patients is not yet well established. In late recurrences, early complete surgical resection should be performed when feasible. Given the high cure rate of TGCT, oncologists should work with patients to prevent and identify potential long-term side effects of the treatment. The above recommendations also apply to extragonadal retroperitoneal and mediastinal tumors.

## Incidence and epidemiology

Testicular cancer is the most frequent tumor in adolescent and young adult males aged between 15 and 39 years [1]. The incidence of testicular germ cell tumors (TGCT) is increasing with more than 74,000 new cases globally per year. The higher incidence is observed in Europe, accounting for almost one third of the cases worldwide. The estimated incidence rate per 100,000 habitants in Europe ranges from 5.0 in Spain to 11.8 in Norway. The 5-year survival of testicular cancer is 96%, including 99.2% for localized tumors, 96% for regional lymph node disease and 73.4% for patients with distant metastases [2], Consequently, all newly diagnosed patients should be treated with curative intent, and therapeutic strategies should minimize acute and long-term side effects.

## Methodology

This guideline is based on a systematic review of relevant published studies and with the consensus of ten treatment expert oncologists from the Spanish Society of Medical Oncology (SEOM) and the Spanish Germ Cell Cancer Group (GG). The Infectious Diseases Society of America-US Public Health Service Grading System for Ranking Recommendations in Clinical Guidelines has been used to assign levels of evidence and grades of recommendation [3]. Table 1 summarizes the main recommendations from SEOM-GG.

**Table 1:** SEOM-GG recommendations for testicular germ cell tumors

SEOM—GG recommendations	Level and grade
Germ cell tumors are curable malignancies; therefore, all newly diagnosed patients should be treated with curative intent, and with therapeutic strategies that minimize acute and long-term side effects	I A
Diagnosis, staging and risk assessment is based on a testicular ultrasound, pre- and post-orchiectomy AFP, BHCG and LDH, and a thoraco-abdominopelvic CT scan. Consider brain imaging in case of extensive metastatic dissemination, very high B-HCG serum levels (>50,000 mIU/ml) or if clinically indicated. Other explorations should be symptoms—guided	III A
Radical inguinal orchiectomy is mandatory to provide local tumor control and to establish the histopathologic subtypes, presence of in situ neoplasia, vascular lymphatic or rete testis invasion and spread beyond the tunica albuginea	III A
Fertility counseling and sperm cryopreservation should be routinely offered if clinically indicated	III A

In patients with stage I testicular germ-cell tumors, both active surveillance and adjuvant chemotherapy are acceptable options following orchiectomy, with no difference in overall survival. Patients should be informed of the pros and cons of the different options, and the final decision should be based on the risk of relapse, patient desires, expectations and wiliness to perform an adequate follow-up	III A
Recommended adjuvant chemotherapy schedules are	
• One course of carboplatin (AUC of 7) for patients with pure seminoma	I A
• One course of BEP for NSGCT	I A
Retroperitoneal lymphadenectomy (for seminoma and NSGCT) and adjuvant radiotherapy (for seminoma) are alternatives not routinely recommended	III A

The IGCCCG prognostic classification remains valid for selecting treatment in advanced disease	III A
• The standard therapy for patients with good prognosis is three cycles of chemotherapy BEP. Four cycles of EP are an alternative for patients with high risk for bleomycin toxicity	I A
• An updated IGCCCG classification has identified additional adverse prognostic factors, including: LDH in disseminated seminomas and LDH, age and pulmonary metastases in disseminated NSGCT. There is currently no evidence on changing treatment recommendations based on this updated classification	III A
• The standard therapy for patients with intermediate and poor risk patients consists of four cycles of BEP chemotherapy. As an alternative, four cycles of VIP can be used when there is an absolute or relative contraindication for bleomycin	I A
Lower dose intensity is associated with poorer outcomes, so it is recommended that full doses are given at the scheduled time, avoiding unnecessary delays and dose reductions wherever possible	I A
Combinations with carboplatin are inferior to combinations with cisplatin and should be avoided unless cisplatin is contraindicated	I A
Tumor marker decline should be monitored in each cycle. Patients with confirmed inadequate marker decline are associated with worse prognosis. This scenario represents an unmet medical need and these patients should be carefully monitored to initiate rescue treatments when necessary	I A
Patients at high risk of relapse and those who have relapsed should be treated by multidisciplinary teams in experienced centers	III A
The above recommendations are also valid for extragonadal retroperitoneal and mediastinal tumors	I A

In patients with NSGCT and post-chemotherapy negative tumor markers, FDG-PET CT is not recommended, and all residual retroperitoneal lymph nodes ≥1 cm or residual masses in other locations should be resected if technically feasible	II A
Conservative follow-up is recommended for seminoma residual masses <3 cm. For residual masses ≥3 cm, the decision should be based on the result of a FDG PET/CT done at least 6 weeks after the last dose of bleomycin. Surveillance is recommended for patients with negative PET-CT. In patients with indeterminate FDG-PET CT a new PET-CT is recommended at 6–8 weeks. In positive tumor masses on PET-CT, consider biopsy followed by resection or salvage treatment if viable residual tumor is confirmed	IIIA

Patients who relapse after stage I should be treated according to the standard recommendations for first-line advanced disease	III A
Patients with advanced GCT who are refractory or who relapse after first-line cisplatin-based chemotherapy should be referred to experienced centers with trained multidisciplinary teams	III A
• Most recommended salvage treatment of these patients include conventional-dose chemotherapy (TIP) and/or high-dose chemotherapy (HDCT), although the best regimen and strategy for each subgroup of patients are not yet well established	III A
• In late relapses, early complete surgical resection must be performed when feasible	III A
Because of most patients with TGCT are cured, oncologists should be aware of potential long-term complications of treatment to advise them with preventive measures and eventually to make an early diagnosis and treatment	III A

*AUC* area under the curve, *AFP* alpha-fetoprotein, *BHCG* beta subunit of human chorionic gonadotropin, *LDH* lactate dehydrogenase, *NSGCT* non-seminomatous germ cell tumors

## Diagnosis, staging and risk assessment

TGCT should be suspected in any male with a solid, painless testicular nodule. A history of cryptorchidism or atrophic testes may be present. Approximately 10% of patients have symptoms of metastatic disease such as lumbar back pain, lower extremity swelling, dyspnea, cough, neck mass enlargement, gynecomastia or paraneoplastic hyperthyroidism. If suspected, diagnosis should be started immediately as any delay in diagnosis may adversely affect tumor stage and prognosis [4].

After a complete physical examination, bilateral high-frequency ultrasound of the testis is required to confirm the presence of a testicular mass and to examine the contralateral testis. The presence of microlithiasis as a single finding is not diagnostic. Other mandatory investigations include a complete blood count and chemistry profile, including pre- and post-orchiectomy STM such as alpha-fetoprotein (AFP), beta-subunit of human chorionic gonadotropin (bHCG) and lactate dehydrogenase (LDH), and a thoraco-abdominopelvic CT scan. Regional metastases first appear in the retroperitoneal lymph nodes, although false-negative results with occult micrometastases may be present in up to 25% of clinical stage I disease. Brain imaging is recommended in patients with extensive pulmonary metastases (i.e., >5 pulmonary nodules), poor IGCCCG risk, very high bHCG levels (i.e., >5000 mIU/ml) or when clinically indicated [5]. Bone scan and/or spinal MRI should be performed if clinical symptoms are present. There is no evidence to support the use of fluorodeoxyglucose PET (FDG-PET) in the staging of testicular cancer [6].

Radical inguinal orchiectomy with ligation of the spermatic cord at the internal inguinal ring is mandatory to facilitate histopathological and prognostic evaluation of the primary tumor and to provide adequate oncologic control [7]. However, in patients with elevated tumor markers and high burden or life-threatening metastatic disease requiring urgent treatment, chemotherapy may be started immediately and orchiectomy delayed until clinical stabilization.

Partial or trans-scrotal biopsy or orchiectomy, are not recommended as they alters the lymphatic drainage to inguinal nodes (“scrotal violation”) [8]. The role of routine contralateral testicular biopsy to exclude germ cell neoplasia in situ (GCNIS, up to 9%), may be discussed in patients with high risk of contralateral GCNIS (i.e., history of cryptorchism and/or testicular volume <12 ml) [9, 10]. Partial orchiectomy for fertility preservation in patients with contralateral tumor (<5%) remain controversial [11].

A pathologic evaluation of the entire testis should be performed instead of a simple biopsy to determine the histopathological subtype according to the latest WHO 2022 histologic classification (Table 2) [12] and the local extent of the disease. Sex cord-stromal tumors of the testis are excluded from this guideline. In practice, germ-cell testicular tumors are classified as seminoma and non-seminomatous germ cell tumors (NSGCT), which include mixed germ cell tumors (GCT). In addition, the presence of in situ neoplasia, vascular or lymphatic or rete testicular invasion, and extension beyond the tunica albuginea or into the spermatic cord are important information for further management and prognosis.

**Table 2:** World Health Organization classification of testicular tumors (WHO, 2022)

Germ cell tumors derived from germ cell neoplasia in situ
Noninvasive germ cell neoplasia
• GCNIS (Germ cell neoplasia in situ)
• Specific forms of intratubular germ cell neoplasia
• Gonadoblastoma
The germinoma family of tumors
• Seminoma
Nonseminomatous germ cell tumors
• Embryonal carcinoma
• Yolk sac tumor, postpuberal type
• Choriocarcinoma
• Placental site trophoblastic tumor
• Epithelioid trophoblastic tumor
• Teratoma, postpuberal type
• Teratoma with somatic-type malignancy
Mixed germ cell tumors of the testis
• Mixed germ cell tumors
Germ cell tumors of unknown type
• Regressed germ cell tumors

Germ cell tumors unrelated to germ cell neoplasia in situ
• Spermatocytic tumor
• Teratoma, prepuberal-type
• Yolk sac tumor, prepuberal type
• Testicular neuroendocrine tumor, prepuberal type
• Mixed teratoma and yolk sac tumor, prepuberal type

Sex cord-stromal tumors of the testis
Leydig cell tumor
• Leydig cell tumor
Sertoli cell tumor
• Sertoli cell tumor
• Large cell calcifying Sertoli cell tumor
Granulosa cell tumor
• Adult granulosa cell tumor
• Juvenile granulosa cell tumor
The fibroma thecoma family of tumors
• Tumors in the fibroma thecoma group
Mixed and other sex cord-stromal tumors
• Mixed sex cord-stromal tumor
• Signet ring stromal tumor
• Myoid gonadal stromal tumor
• Sex cord-stromal tumor NOS

Tumor markers (AFP, bHCG, and LDH) should be performed before surgery as they support diagnosis of TGCT and may be indicative of subtype. However, they have low sensitivity and normal values do not exclude TGCT. AFP and/or bHCG are elevated in about 85% of NSGCTs, even in localized tumors. By contrast, serum bHCG is elevated in less than 20% of testicular seminomas, and AFP is not elevated in pure seminomas where an increase of AFP indicates a non-seminoma component.

Serum tumor markers should be closely monitored after orchiectomy. A progressive decline to normalization according to their half-lives (5–7 days for AFP and 1–3 days for bHCG) confirms that orchiectomy has removed all tumor disease, otherwise they provide early evidence of residual disease or recurrence.

TGCT are staged using the eighth (2016) tumor, node, metastasis (TNM) staging system developed jointly by the American Joint Committee on Cancer and the Union for International Cancer Control, based on imaging and STM after orchiectomy (Table 3) [13]. Localised tumor includes T1-4N0M0S0, all others should be considered as disseminated disease, including those patients without radiologic evidence of metastasis whose tumor markers do not normalize after orchiectomy. Advanced stages (IS-III) are further classified according to the IGCCCG prognostic model (Table 4).

**Table 3:** Testicular cancer TNM staging AJCC UICC 8th edition

Primary tumor (T)	
pTx	Primary tumor cannot be assessed
pT0	No evidence of primary tumor
pTis	Germ cell neoplasia in situ
pT1	Tumor limited to testis (including rete testis invasion) without lymphovascular invasion
	Subclassification of pT1 applies only to pure seminoma:
pT1a	Tumor smaller than 3 cm in size
pT1b	Tumor 3 cm or larger in size
pT2	Tumor limited to testis (including rete testis invasion) with lymphovascular invasion or tumor invading hilar soft tissue or epididymis or penetrating visceral mesothelial layer covering the external surface of tunica albuginea with or without lymphovascular invasion
pT3	Tumor directly invades spermatic cord soft tissue with or without lymphovascular invasion
pT4	Tumor invades scrotum with or without lymphovascular invasion

Regional lymph nodes (N)	
Nx	Regional lymph nodes cannot be assessed
N0	No regional lymph node metastasis
N1	Metastasis with a lymph node mass 2 cm or smaller in greatest dimension or multiple lymph nodes, none larger than 2 cm in greatest dimension
N2	Metastasis with a lymph node mass larger than 2 cm but not larger than 5 cm in greatest dimension or multiple lymph nodes, any one mass larger than 2 cm but not larger than 5 cm in greatest dimension
N3	Metastasis with a lymph node mass larger than 5 cm in greatest dimension

Distant metastasis (M)	
M0	No distant metastases
M1	Distant metastases
M1a	Non retroperitoneal nodal or pulmonary metastases
M1b	Non pulmonary visceral metastases

Serum markers postorchiectomy (S)	
Sx	Marker studies not available or not performed
S0	Marker study levels within normal limits
S1	LDH < 1.5 × ULN and hCG (mIU/ml) < 5000 and AFP (ng/ml) < 1000
S2	LDH 1.5–10 × ULN or hCG (mIU/ml) 5000–50,000 or AFP (ng/ml) 1000–10,000
S3	LDH > 10 × ULN or hCG (mIU/ml) > 50,000 or AFP (ng/ml) > 10,000

TNM prognostic stage groups	
Stage 0	pT0 N0 M0 S0
Stage I	pT1-4 N0 M0 Sx
Stage IA	pT1 N0 M0 S0
Stage IB	pT2-4 N0 M0 S0
Stage IS	Any pT N0 M0 S1-3
Stage II	Any pT N1-3 M0 Sx
Stage IIA	Any pT N1 M0 S0-1
Stage IIB	Any pT N2 M0 S0-1
Stage IIC	Any pT N3 M0 S0-1
Stage III	Any pT Any N M1 Sx
Stage IIIA	Any pT Any N M1a S0-1
Stage IIIB	Any pT Any N M0-1a S2
Stage IIIC	Any pT N1-3 M0 S3, any pT Any N M1a S3 or any pT Any N M1b Any S

*TNM* tumor, node, metastasis; *AJCC* American Joint Committee on Cancer; *UICC* Union for International Cancer Control; *LDH* lactate dehydrogenase; *hCG* human chorionic gonadotropin; *AFP* alpha-fetoprotein

**Table 4:** Risk stratification system for advanced testicular germ cell tumors: International Germ Cell Cancer Collaborative Group (IGCCCG) 1997

	Seminoma	Nonseminomatous germ cell tumors
Good risk	Testicular or extragonadal origin + No metastases outside lungs and/or lymph nodes + Any BHCG or LDH value	Testicular or retroperitoneal origin + No metastases outside lungs and/or lymph nodes + BHCG < 5000 mIU/ml + AFP < 1000 ng/ml + LDH < 1.5 × ULN
Intermediate risk	Testicular or extragonadal origin Metastases to organs other than lungs or lymph nodes + Any BHCG or LDH value	Testicular or retroperitoneal + No metastases outside lungs and/or lymph nodes + BHCG 5000–50,000 mIU/ml or AFP 1000–10,000 ng/ml or LDH 1.5–10 × ULN
Poor risk		Mediastinal primary or Metastases to organs other than lungs or lymph nodes or BHCG > 50,000 mIU/ml or AFP > 10,000 ng/ml or LDH > 10 × ULN

Markers used for staging and risk classification are postorchiectomy

See text for additional prognostic information

*AFP* alpha-fetoprotein, *BHCG* beta-human chorionic gonadotropin, *LDH* lactic dehydrogenase, *mU/ml* milli-international units/ml, *ng* nanograms

Infertility or impaired spermatogenesis is common in patients with testicular cancer before the start of treatment [14], but can be exacerbated by orchiectomy, cisplatin-based chemotherapy or radiotherapy. Approximately 70% of patients will recover spermatogenesis, depending on age, type of treatment and severity of previous oligospermia. Information and counselling on fertility issues and sperm cryopreservation should be offered routinely prior to the initiation of any form of treatment, ideally prior to orchiectomy.

## Management of localized testicular germ-cell tumors

### Stage I seminoma

Approximately 80% of patients with seminoma present with stage I disease, which is associated with a long-term survival rate of 99%. Recurrences on surveillance are uncommon (15–20%), occur in the first 14–18 months, mainly in the retroperitoneum, and are highly curable with cisplatin-based chemotherapy [15]. Tumor size (TS), considered as a continuous variable, stromal rete testis invasion (RTI) and lymphovascular invasion (LVI) are the main predictive factors for relapse on surveillance. Relapse-free survival in patients with TS ≤ 5 cm without RTI or LVI or TS ≤ 2 cm with either RTI or LVI is 89–94%, in contrast to 34–73% in those with TS > 5 cm and both RTI and LVI, and 76–84% in the remaining patients [16].

Therapeutic options after orchiectomy should be discussed with the patient. Active surveillance is the preferred strategy for most patients, but adjuvant chemotherapy with a single course of carboplatin (area under the curve of 7) is an alternative, especially for those with more than one risk factor or for those unwilling or unable to undergo surveillance [17, 18]. Some non-randomised studies suggest that two cycles of carboplatin may be associated with a lower risk of relapse, but there is limited data on the long-term toxicities of carboplatin [19]. Due to the increased risk of second malignancies, low-dose reduced paraaortic adjuvant radiotherapy should only be recommended if chemotherapy is contraindicated [20].

### Stage I NSGCT

Approximately two thirds of patients with NSGCT are diagnosed with stage I disease. Orchiectomy alone cures about 75% of these patients. The remainder will relapse, usually within the first 2 years after surgery, the majority as good risk advanced disease. The presence of LVI in the primary tumor defines a subgroup with a high risk of relapse, approaching 50% (as opposed to 15% in the remaining patients). Predominance of embryonal carcinoma is also associated with an increased recurrence rate. The expected relapse rates are 25%, 41% and 77%, respectively, when none, one or both of these factors are present [21].

The 5-year disease-specific survival of patients with stage I NSGCT is close to 100%, regardless of the postoperative strategy. Active surveillance of all patients provides an excellent cure rate, avoiding unnecessary therapy and potential long-term toxicity in many patients. Alternatively, a risk-adapted approach, i.e., the administration of adjuvant chemotherapy to high-risk patients, allows for less intensive follow-up, reducing the associated stress and disruption of life and reducing the need for post-chemotherapy retroperitoneal lymphadenectomy in the event of recurrence. Based on a prospective non-randomized study, one cycle of standard BEP chemotherapy (Table 5) reduces the risk of relapse to less than 5% in patients with LVI and is the most commonly recommended adjuvant treatment for patients with LVI [22]. Retroperitoneal lymphadenectomy is reserved for selected patients with LVI, contraindications to adjuvant BEP and doubtful ipsilateral lymph nodes on CT scan [23].

**Table 5:** First-line and salvage chemotherapy schedules for IGCCCG advanced testicular germ cell tumors (stages IS-III)

First line		Salvage treatment		
IGCCCG good risk	IGCCCG intermediate or poor risk	After AS, RPLND, RT or adjuvant CT	Failure to first line	Failure to second line
First choice: BEP × 3 Alternative: EP × 4	First choice: BEP × 4 Alternative: VIP × 4	Similar to standard first line as per IGCCCG^a^	Conventional CT (TIP or VeIP), or HDCT	HDCT after TIP or VeIP Clinical trial Palliative CT^b^

Chemotherapy regimens^c^	
Carboplatin	Total dose AUC 5
BEP	Bleomycin 30 units on days 1, 8, and 15 Etoposide 100 mg/m^2^/day on days 1–5 Cisplatin 20 mg/m^2^/day on days 1–5
EP	Etoposide 100 mg/m^2^/day on days 1–5 Cisplatin 20 mg/m^2^/day on days 1–5
BEC	Bleomycin 30 units on day 2 Etoposide 120 mg/m^2^/day on days 1–3 Carboplatin AUC 5 on day 1
VIP	Etoposide 75 mg/m^2^/day on days 1–5 Cisplatin 20 mg/m^2^/day on days 1–5 Ifosfamide 1200 mg/m^2^/day days 1–5. Mesna 120 mg/m^2^ iv bolus on day 1 before ifosfamide, then 1.2 g/m^2^/day continuous infusion on days 1–5
VeIP	Vinblastine 0.11 mg/kg/day, days 1 and 2 Cisplatin 20 mg/m^2^/day on days 1–5 Ifosfamide 1200 mg/m^2^/day days 1–5. Mesna 120 mg/m^2^ iv bolus on day 1 before ifosfamide, then 1.2 g/m^2^/day continuous infusion on days 1–5
TIP^d^	Paclitaxel 250 mg/m^2^ continuous infusion over 24 h day 1 Cisplatin 25 mg/m^2^/day on days 2–5 Ifosfamide 1500 mg/m^2^/day days 2–5. Mesna 120 mg/m^2^ iv bolus on day 1 before ifosfamide, then 1.5 g/m^2^/day on days 2–5 Pegfilgrastim on days 6 or 7 or filgrastim daily from day 7 to 18 or until neutrophil recovery

*AS* active surveillance, *AUC* area under the curve *CT* chemotherapy, *HDCT* high-dose chemotherapy, *IGCCCG* International Germ Cell Cancer Collaborative Group, *RPLND* retroperitoneal lymph node dissection, *RT* radiotherapy

^a^Cumulative dose of bleomycin should not exceed 360 UI

^b^Palliative CT includes Paclitaxel-Gemcitabine, Oxaliplatin-Gemcitabine or oral etoposide

^c^Cycles every 21 days. See text for references

^d^Doses selected for the TIGER trial

## Management of advanced and metastatic disease

### General recommendations

A validated prognostic model for advanced disease was developed by the International Germ Cell Cancer Collaborative Group (IGCCCG). Patients with advanced disease (stages IS, II and III) were classified into good, intermediate, and poor risk groups for both progression-free and overall survival, based upon histology (seminoma vs. nonseminoma), primary site of the tumor, metastatic sites, and STM levels (Table 4) [24].

This classification remains valid and is the basis for selecting appropriate treatment, although it has recently been updated to include modern treatments and longer follow-up [25, 26]. For patients with disseminated seminoma, the expected 5-year OS is 95% and 88% for good and intermediate prognosis, respectively, although the prognosis of those with good prognosis with LDH > 2.5 × ULN is very similar to that of those with intermediate risk. For patients with advanced NSGCT, the 5-year OS is 96%, 89% and 67% for good, intermediate and poor prognosis respectively. However, in the latest update, a more refined prognostic model was developed and validated, including LDH > 2.5 × ULN, age and the presence of pulmonary metastases as additional adverse prognostic factors.

Advanced disease includes stages IS to III. Cisplatinum-based chemotherapy is the cornerstone of systemic treatment for germ cell cancer (Table 5). Bleomycin, etoposide and cisplatin (BEP) is the standard of care. Patients with intermediate- and poor-risk IGCCCG should be treated with four cycles of BEP, whereas patients with good-risk IGCCCG can be safely treated with three cycles of BEP [27].

An absolute or relative contraindication to bleomycin may exist in patients over 40 years of age, those with pulmonary disease, heavy smokers, athletes or professionals who require a high lung capacity, and those with mediastinal tumors or lung metastases, especially if extensive pulmonary resection or radiation is planned after chemotherapy [28]. Baseline and follow-up spirometry and diffusing capacity for carbon monoxide (DLCO) may identify these patients as ineligible for bleomycin, as well as early toxicity during treatment. Bleomycin toxicity should be suspected in any patient with sudden onset of cough or dyspnea. A decrease in corrected DLCO is a predictor of bleomycin-induced pneumonitis. We recommend discontinuing bleomycin if a decrease in DLCO greater than 25% is observed [29].

If there is a contraindication to bleomycin in patients with IGCCCG good prognosis tumors, four cycles of EP [30] may be used as an alternative, although slightly statistically non-significant worse results have been reported in two randomised trials in NSGCT [31, 32]. For patients with advanced IGCCCG intermediate or poor prognosis tumors, the alternative first line schedule is VIP (Table 5) plus prophylactic G-CSF [33, 34]. Combinations of carboplatin with etoposide (EC) in patients with good prognosis or with bleomycin and etoposide in NSGCT (BEC) are inferior to the same combinations with cisplatin [35, 36]. Radiotherapy (30 Gy) on the retroperitoneal ipsilateral and iliac lymph nodes could also be an alternative for selected stage IIA and IIB patients with seminoma who refuse or have contraindication for chemotherapy [37, 38].

BEP is generally well tolerated, especially in patients with a good prognosis, although many patients may experience myelosuppression (especially neutropenia), fatigue, alopecia and, in some, nausea, peripheral neuropathy, tinnitus or hearing loss, and even renal and pulmonary toxicity. The oncologist should aim to administer the full dose at the scheduled time, avoiding delays and dose reductions as much as possible, as lower dose intensity is associated with worse outcomes [39]. We recommend prophylactic G-CSF to achieve these goals. Because of the low haematological toxicity of bleomycin, it can generally be given on days 8 and 15 of each cycle, even if the blood-cell count is low, although the dose should be adjusted in patients with a creatinine clearance <50 ml/min and discontinued in the event of pulmonary toxicity. In any case, the total cumulative dose should not exceed 360–400 UI. A dose reduction of etoposide or ifosfamide should be considered in the event of prolonged febrile neutropenia, incomplete blood-cell recovery, bleeding or G4 hematologic toxicity in the previous cycle [40].

Tumor marker decrease should be monitored before each cycle. The Spanish Germ Cell Cancer Group Registry has a serum tumor marker calculator available to all members (www.grupogerminal.es). Tumor marker decline is the only confirmed prospective predictor of response to chemotherapy in patients with metastatic germ cell cancer. Patients with inadequate decline after the first or second cycle represent a group with a poorer prognosis. The GETUG-13 trial showed that patients with a favorable tumor marker response after one cycle of BEP are likely to be cured in more than 80% of cases if BEP is continued [41]. Patients with inadequate tumor marker response represent an unmet medical need where close monitoring and early salvage strategies should be considered [42].

Importantly, these guidelines recommend that patients with TGCT at high risk of recurrence, as well as those who have relapsed, be treated by multidisciplinary teams in experienced centers [43].

### Special situations

#### Extragonadal germ cell tumors

Extragonadal GCT are rare neoplasms (1–5% of all GCTs) that originate in midline locations such as mediastinum or retroperitoneum, probably from primordial germ cells that fail to migrate to the gonadal ridges during embryonal development [44]. Sacrococcygeal and intracranial GCTs, most common in children and adolescents, are not covered in this guide. Histologic diagnosis and an accurate differential diagnosis with other histologies, such as thymic carcinomas and lymphomas is encouraged.

Retroperitoneal GCTs have a similar clinical presentation, prognosis and treatment as disseminated testicular tumors, although they are usually bulky at diagnosis because they are oligosymptomatic in their early stages. Treatment is based on systemic cisplatin-based chemotherapy following the recommendations above for each of the IGCCCG subgroups, as well as the management of residual disease described below [45].

Primary mediastinal GCT have different molecular and clinical features compared to TGCT. Although the prognosis depends on the extent, it appears to be similar to that of TGCT for seminomas and worse for NSGCT. The treatment of mediastinal tumors generally requires a multimodality approach. Chemotherapy is usually given first followed by surgery to remove any residual masses, although the optimal order of these therapies has not been established. Chemotherapy BEP, EP or VIP should be chosen according to the above general recommendations for each IGCCCG prognostic subgroup, balancing the ability to control disease while minimizing the risk of bleomycin toxicity, taking into account the possibility of future mediastinal surgery and the potential need for partial lung resection. Most patients with mediastinal NSGCT have residual masses at the end of chemotherapy. Removal of all residual masses after chemotherapy plays an important role in the treatment of these tumors and should be performed whenever technically possible [46]. For mediastinal seminomas, radiotherapy may be an alternative in patients with contraindications to surgery.

#### Management of post-chemotherapy residual disease

Decisions on residual masses after completion of chemotherapy should be made based on the initial histology, location of the residual lesions, and the evolution of tumor markers.

Patients with NSGCT, post-chemotherapy negative tumor markers and residual retroperitoneal lymph nodes ≥1 cm in larger axial diameter should undergo surgery, preferentially an open nerve-sparing retroperitoneal lymph node dissection (RPLND). In large residual masses, a full bilateral RPLND is recommended, whereas a modified template RPLND can be considered in cases of low volume before and after CT [47]. FDG-PET-CT is not recommended for the evaluation of residual disease in NSGCT. Pathologic examination of RPLND following chemotherapy demonstrate necrosis in 50% of cases, mature teratoma in 35%, and viable tumor in 15%. Persistent intrathoracic masses as well in other locations should be resected if technically feasible [48]. Although the timing for metastasectomy is not well established, the retroperitoneum is commonly selected as the initial site for resection due to its higher frequency of residual disease. However, pathologic discrepancy between retroperitoneal lymph node and thoracic residual masses is about 30%. Pathologic concordance between the two lungs is greater than 90% [49]. Thus, patients with necrosis in both retroperitoneum and in one side of the lung can avoid contralateral lung surgery [50].

In contrast, active surveillance is recommended for patients with disseminated seminoma and post-chemotherapy residual disease with a larger diameter less than 3 cm. In the rest of patients, a FDG PET/CT should be done at least 6 weeks after the last dose of bleomycin. In case of negative FDG-PET, we recommend active surveillance due to its high negative predictive value (>90%). In case of indeterminate results, we recommend repeating a new PET/TC 8–12 weeks later, due to its limited positive predictive value. If FDG-PET is unequivocally positive, we recommend resection of the residual mass, but due to the limited positive predictive value of FDG-PET, and the difficulty and morbidity of resection of residual masses in seminoma, which often have an associated desmoplastic reaction, some authors propose a biopsy of the lesion to confirm tumor persistence before making a therapeutic decision. Radiotherapy may be an option if residual disease is confirmed and resection is not feasible.

#### Postoperative chemotherapy after resection of residual disease

Despite postoperative treatment has not demonstrated to increase overall survival and is controversial, two additional cycles of chemotherapy (EP, VIP or TIP) are commonly recommended for patients with more than 10% of viable tumor in the residual mass, particularly if they were of intermediate or poor-risk disease and/or they had incomplete resection [51].

#### Choriocarcinoma syndrome and patients at risk of acute respiratory distress syndrome (ARDS)

Choriocarcinoma is a highly vascularized tumor with rapid development of extensive metastasis particularly in the lung, but also in the liver, brain, and other organs. Because bleeding leading to ARDS and other severe complications may even be triggered with the first standard cycle of chemotherapy, an initial dose-reduced induction regimen such as a 2- or 3-day EP [52, 53]or baby-BOP (Cisplatin 50 mg/m^2^, vincristine 2 mg, and bleomycin 30 U on day 1) [54] has been recommended. After 14 days of this regimen once the patient is stabilized the full number of cycles should be applied following the induction cycle. If induction EP was used, the remaining additional days of the EP protocol may be administered at day 15 when clinically feasible, before starting standard BEP. Orchiectomy should be performed in all patients with testicular lesions, but if the patient is not stable at the time of diagnosis, chemotherapy should be started and orchiectomy delayed even until the end of systemic treatment.

These induction approaches are also valid for other patients with NSGCT and at high risk of ARDS due to extensive lung metastases, dyspnea or hypoxemia at diagnosis.

In cases of extensive tumor volume, prevention measures for tumor lysis syndrome is also necessary.

#### Patients unfit for cisplatin

Patients who are definitely unfit for cisplatin-based CT can be treated with carboplatin-based chemotherapy, although results are inferior to BEP [35, 36]. In patients with obstructive uropathy, a nephrostomy before initiating CT should be performed to be able to administer cisplatin.

#### Brain metastases

Brain metastases occur in about 10% of patients with advanced disease, either in the context of initial metastatic disease, as a part of a systemic relapse or rarely as an isolated site of relapse. Long-term survival of patients presenting with brain metastases at diagnosis is poor (30–50%) and even poorer when a site of recurrent disease (5-year survival rate is 2–5%) [5]. Brain metastases usually require a multimodal approach, although the optimal sequence should be individualized.

The general approach for patients with brain metastases is chemotherapy followed by observation in case of complete response, or surgical excision and/or stereotactic radiosurgery in case of small residual disease. In patients with brain metastases at relapse, consolidation RT should be used, even with total response after chemotherapy. Surgery should be considered in case of a persistent solitary metastases but location of metastases, histology of primary tumor and systemic disease status should be considered. Palliative whole-brain radiation therapy is indicated in multiple unresectable lesions [55].

#### Prophylaxis of thromboembolic events (TEE)

TEE occur more frequently in GCT patients receiving chemotherapy than in patients of the same age receiving chemotherapy for other cancers. Retrospective studies identified increasing stage and size of retroperitoneal lymph nodes, as well as Khorana score and indwelling vascular access devices as TEE risk factors [56]. Data regarding the efficacy of thromboprophylaxis are conflicting [57] but despite lacking level-I evidence, prevention of TEE should be particularly considered in patients at higher risk, such as those with retroperitoneal involvement >3.5 cm, stage III or poor prognosis IGCCCG [58]. In addition, vascular access devices should be avoided whenever possible.

#### Growing teratoma syndrome

This is a rare condition associated with NSGCT, characterized by an increase in metastatic mass during or after chemotherapy with normalized STM, caused by a mature teratoma with no malignant component. Treatment consists of surgical resection of the lesions [59].

#### Teratoma with malignant transformation (TMT)

TMT into somatic histologies is a rare but significant complication that occurs in less than 6% of metastatic GCTs. This transformation results in the emergence of a variety of non-germ cell histologies, such as adenocarcinoma, squamous cell carcinoma, sarcoma, and others, which may coexist with the original germ cell tumor. When present at metastatic sites, TMT is associated with a poor prognosis. Somatic type of malignancy, grade, extent of disease, feasibility of radical surgery, number of prior chemotherapy lines of treatment, and the primary tumor site had been also proposed as determinants of long-term outcomes [60, 61].

As these tumors are often resistant to standard platinum-based chemotherapy and radiotherapy, their management remains a challenge for clinicians. The most effective therapeutic approach currently available is complete resection, which often requires aggressive and extensive resection, especially when the disease is confined to solitary sites. Adjuvant chemotherapy as well as systemic treatment when complete resection is not possible, should be individualized and tailored to the transformed histology, particularly in sarcomas and primitive neuroectodermal malignant transformation [62].

## Treatment of relapsed and refractory tumors

Patients with stage I disease at diagnosis followed by surveillance, RPLND, radiotherapy, carboplatin, and even those who have received one cycle of BEP, should be treated at relapse according to standard recommendations for first-line advanced disease, but taking into account the previous cumulative dose of bleomycin. RPLND may be an option in patients with NSGCT if teratoma is suspected (depending on the presence of tumor markers and the extent of disease at relapse) [63].

Approximately 18–20% of patients with advanced TGCT are refractory or relapse after first-line chemotherapy and require additional salvage therapies, with 5-year PFS rates ranging from 54 to 90% depending on IGCCCG subgroups. These patients are still potentially curable, albeit in a much smaller proportion than first-line patients, and should preferably be treated by experienced teams in reference centres.

Patients who fail first-line cisplatin-based chemotherapy should be classified according to the International Prognostic Factor Study Group (IPFSG) classification, a risk prognostic model based on a large retrospective series (Table 6). The IPFSG established five prognostic categories based on primary site, previous response to therapy, progression-free interval, tumor marker levels, histology, and presence of metastases in the liver, bone or brain. Two-year progression-free survival rates ranged from 75% in the very low-risk group to 6% in the very high-risk subgroup [64].

**Table 6:** The International Prognostic Factor Study Group (IPFSG)

	Score points			
	0	1	2	3
Primary	Gonadal	Extragonadal		Mediastinal non seminoma
Prior response	CR/PRm−	PRm+/SD	PD	
PFI	>3 months	≤3 months		
AFP	Normal	≤1000	>1000	
HCG	≤1000	>1000		
LBB	No	Yes		
Score sum (values 0–10)				
Regroup score sum into categories: (0) = 0; (1 or 2) = 1; (3 or 4) = 2; (5 or more) = 3				
Add histology score points: seminoma = −1; non seminoma or mixed tumors = 0				
Final prognostic score				
Very low = −1; low = 0; intermediate = 1; high risk = 2; very high risk = 3				

*CR* complete response, *PRm*− partial response markers negative, *PRm*+ partial response markers positive, *SD* stable disease, *PD* progression disease, *PFI* platinum-free interval, *AFP* alpha-fetoprotein at salvage treatment, *HCG* human chorionic gonadotrophin at salvage treatment, *LBB* liver, bone, or brain metastasis

There are two main options for salvage treatment of these patients: conventional dose chemotherapy (CDCT), and high dose chemotherapy (HDCT). Approximately one third of patients treated with one of these regimens become long-term survivors. Although the best regimen and strategy for each IPFSG patient subgroup is not yet well known, retrospective analyses suggest that HDCT may be superior to CDCT in the majority of patients, but the toxicity associated with HDCT can be significant and its benefit has not been clearly demonstrated. The results of the large randomised phase III TIGER trial comparing the two strategies are eagerly awaited. In the meantime, both options are considered valid. It is important to note that surgery of all residual lesions after chemotherapy must be performed in all cases if technically feasible, regardless of the type of treatment administered [65].

In the CDCT approach, the most commonly used salvage regimen after BEP is four cycles of TIP with GCSF support [66–68]. The regimen VeIP and VIP, with vinblastine or etoposide respectively instead of paclitaxel, could be an alternative in some patients [69] (Table 4). Two main strategies of HDCT are available for these patients. One of them consists of two cycles of HDCT with carboplatin and etoposide with autologous peripheral-blood hematopoietic stem cells support preceded by one or two cycles of standard-dose chemotherapy with VeIP or VIP, that are used for leukapheresis of peripheral-blood stem cells [70]. The other approach is the TICE regimen, that included two cycles of paclitaxel plus ifosfamide with leukapheresis, followed by three cycles of high-dose carboplatin plus etoposide with reinfusion of peripheral-blood stem cells [71].

Some patients who progress after CDCT can be rescued with HDCT as a second or subsequent salvage therapy. In the remainder of patients, including those who progress after HDCT, subsequent lines are usually palliative and only occasionally lead to long-term survival. Clinical trials, including early-phase trials, should be prioritized in this scenario. Treatments commonly used in patients who progress after HDCT and when a clinical trial is not an option include: paclitaxel–gemcitabine, oxaliplatin–gemcitabine (GEMOX) or oral etoposide. Some patients progressing after CDCT can be rescued with HDCT as second or subsequent salvage therapy. In the rest of patients, including those who progress after HDCT, subsequent lines are usually palliative, and only occasionally they result in long-term survival. Clinical trials should be prioritized in this scenario, including early-phase clinical trials. Treatments commonly used in patients in progression to HDCT and, when a clinical trial is not an option, include: paclitaxel–gemcitabine, oxaliplatin-gemcitabine (GEMOX) or oral etoposide [72].

Late relapse after first-line chemotherapy, defined as tumor recurrence more than 2 years after primary systemic treatment, represents a special situation characterized by a higher degree of resistance to chemotherapy. In these cases, early complete surgical resection is the mainstay of treatment whenever possible. However, salvage chemotherapy is usually also required in conjunction with surgery [73].

## Follow-up

Given the good treatment outcomes of TGCT, a large population of young long-term survivors is to be expected. These patients require an appropriate follow-up program that balances efficacy to detect relapses early, without an excessive burden of visits to facilitate adherence and with as little radiation exposure as possible related to imaging tests. In recent years, there has been increasing interest in adopting less intensive imaging strategies, especially in stage I tumors. The following paragraphs and Table 7 summarize the SEOM-Grupo Germinal recommendations based on the most recent evidence and compiling endorsements from other groups with broad expertise in the management of this disease [74]. It is important to notice that no single follow-up plan is appropriate for all patients, and the following recommendations are to provide guidance, and should be adapted to each individual patient.

**Table 7:** Follow-up of testicular germ cell tumors after initial treatment

Stage	Treatment applied	Exams to perform	1st year	2nd year	3rd year	4th year	5th year	Beyond year 5
*Follow-up schemas for seminomas*								
I	Surveillance^a^	H&P ± STM^b^	q. 4–6 months	q. 6 months	q. 6 months	Once a year	Once a year	Individualize^d^
		AbdP CT/MRI^a,b^	q. 4–6 months	q. 6 months	q. 6 months	Once a year	At month 60	
		Chest CT^c^	Only if clinically indicated					
I	Adjuvant Carbo	H&P ± STM^a^	q. 6 months	q. 6 months	Once a year	Once a year	Once a year	Individualize^d^
		AbdP CT/MRI^b^	q. 6 months	Once a year	Once a year	N/A	At month 60	
		Chest CT^c^	Only if clinically indicated					
II–III	Chemotherapy^e^	H&P ± STM	q. 3 months	q. 3 months	q. 6 months	q. 6 months	Once a year	Individualize^d^
		AbdP CT	q. 6 months	q. 6 months	Once a year	N/A	At month 60	
		Chest CT [only if previous lung or supradiaphragmatic metastases]	q. 6 months	q. 6 months	Once a year	N/A	At month 60	
*Follow up schemas for non-seminomas*								
I	Surveillance	H&P + STM	q. 2 months	q. 3 months	q. 4 months	Once a year	Once a year	Individualize^d^
		AbdP CT/MRI^b^	q. 4 months	q. 6 months	N/A	N/A	At month 60	
		Chest CT^c^	q. 4 months	q. 6 months	N/A	N/A	At month 60	
I	Adjuvant BEP	H&P + STM	q. 3 months	q. 3 months	q. 6 months	q. 6 months	Once a year	Individualize^d^
		AbdP CT/MRI^b^	q. 6 months	Once a year	Once a year	N/A	At month 60	
		Chest X ray	q. 6 months	Once a year	Once a year	N/A	At month 60	
II–III	Chemotherapy^f^	H&P + STM	q. 2 months	q. 3 months	q. 6 months	q. 6 months	Every 6 months	Individualize^e^
		AbdP CT	q. 6 months	q. 6 months	Once a year	Once a year	Once a year At month 60	
		CT chest [only if previous lung or supradiaphragmatic metastases]	q. 6 months	q. 6 months	Once a year	Once a year	At month 60	

*AbdP* abdominopelvic, *CT* computed tomography scan, *H&P* history and physical examination, *N/A* not applicable, *STM* serum tumor markers

^a^Frequency of tests and visits can be individualized and shorten (4 months vs. 6 months) in those patients considered at high risk of relapse based on tumor size, and rete testis or lymph vascular invasion

^b^Physical examination and tumor markers have low probability of capture a relapse in Clinical Stage I Seminomas (CSIS), and therefore are considered optional. MRI has been shown to be non-inferior to CT, and it is recommended as an alternative to decrease radiation exposure

^c^Imaging of the chest is NOT routinely recommended in follow up of CSIS, but it is in CSINS. When needed consider LOW DOSE CT

^d^After 5 years of adequate follow-up, discharge to primary care can be considered or alternatively (as suggested by high volume centers like Princess Margaret Cancer Centre) continued with yearly follow-up until 10 years including imaging (CT or MRI) at years 7 and 9

^e^After 5 years of adequate follow-up, discharge to primary care can be considered although individual cases might be considered for yearly clinical controls. In patients who received chemotherapy, either in the adjuvant setting or in the context of advanced disease is recommended to maintain yearly follow-up to control/identify late toxicities. Blood work including lipid and glycemic profiles ± testosterone are recommended

^f^This category applies only to patients with Stages II or III and favorable response after chemotherapy [either complete or partial response with residual mass <1cm or resected]. Patients with complete tumor marker response but unresectable residual masses >1 cm should have a closer follow-up particularly if teratoma in the primary tumor. Follow-up of metastases in particular locations [i.e., brain or bone] requires specific imaging tests

### Clinical Stage I Seminomas (CSIS)

Cure rates for CSIS are close to 100% regardless of the initial approach, which includes either surveillance or adjuvant carboplatin after orchidectomy. Recurrences occur in approximately 6–20% and 3–6% after surveillance and adjuvant carboplatin, respectively. Most of these relapses (75–95%) are observed within the first 2–3 years and >95% within 5 years, with a median time to relapse of 14–21 months. In terms of location, most patients (90%) relapse in the retroperitoneum, and therefore cross-sectional imaging is the main means of detection. Conversely, the frequency of recurrences detected exclusively by other methods is anecdotal, as only 0–5%, 0%, and 5–10% are diagnosed by clinical examination, chest x-ray, or serum tumor marker, respectively [15].

These observations have shaped over the years the follow-up recommendations. The SEOM-Grupo Germinal proposal for CSIS is to adapt the follow-up schedule according to the treatment option utilized in this clinical setting that conditions the risk of recurrence [i.e., active surveillance or adjuvant carboplatin]. Although physical exam and serum tumor markers (STM) are also included in the recommendations the critical component is the cross-sectional imaging of the abdomen and pelvis as more than 90% of the relapses will occur in the retroperitoneum. In general, for patients who opted for active surveillance, imaging of the abdomen and pelvis is recommended every 6 months for the first 3 years and then annually in years 4 and 5. In the other hand for those patients who received adjuvant carboplatin imaging of the abdomen and pelvis is recommended less intensively, every 6 months only the first year and then annually on years 2 and 3, omit year 4 and perform an imaging test at the end of year five. Contrary to our previous guideline, imaging of the chest is no longer routinely recommended. After 5 years, follow-up needs to be individualized as no consensus exist in the literature. Testicular ultrasound should be considered in years 3 and 5 in the presence of a normal contralateral testis, or more frequently in patients with risk factors or previous abnormal ultrasound findings such as microcalcifications.

### Clinical Stage I NSGCT

General follow-up recommendations should be individualized according to the presence or absence of factors that increase risk of recurrences and treatment received. For patients who opt for exclusively active surveillance and no treatment intervention, we recommend a more intense follow-up. Thus, during the first year when the risk of recurrence is the highest every 2 months visits with STM and quarterly imaging tests are recommended. The second-year frequency of visits can be extended to every 3 months with imaging performed only every 6 months. Given the rarity in CSINS patients of relapses beyond 2 years no cross-sectional imaging is recommended in years 3 and 4 where visits with STM will be every 4 months and once a year respectively. During year 5, yearly visits with a final imaging evaluation in month 60 is recommended.

For those patients who opted for adjuvant BEP the frequency of visits and STM is less intense recommending every 3 months during the first 2 years and then switching to every 6 months in years three and four and yearly in year five. Cross-sectional imaging likewise is recommended with less frequency reducing the total number of tests in this group and therefore an imaging test is recommended every 6 months in year one and then yearly in years 2 and three, omitting year four and performing an imaging test at the end of year five.

After 5 years, follow-up needs to be individualized both for CSIS and CSINS as no consensus exist in any of the two groups.

### Advanced seminoma

When advanced disease, overall benefit in patients with seminoma after chemotherapy is high with around two thirds of patients achieving a favorable response including 30% of complete responses. It is estimated than less than 20% of patients experience relapse after systemic treatment with a median of 9 months with the retroperitoneum and lung as the most common relapse sites with 90% and 10% respectively [75].

This relapse profile defines the current follow-up recommendation that changes in comparison with NSGCT with less frequent visits but longer imaging follow-up and with variable evaluation of the chest as summarized in Table 7.

### Advanced NSGCT

After achieving a favorable response, it is estimated that around 20% of patients with NSGCT might relapse. Recurrences differ from seminomas in shorter timing [median time to relapse of 3 months and most relapses within the first 2 years], broader location [retroperitoneum (33%), pelvis (25%) and lung (33%)], and value of STM [three quarters of recurrences can be detected by TM] [76]. All these particularities lead to a slightly different follow up schema that is illustrated in Table 7.

### Additional recommendations

Testicular ultrasound and self-examination should be included in the follow-up. Approximately 1–5% of patients with a prior history of testicular cancer will develop a contralateral testicular cancer in the next 20 years, with >25% of metachronous TGCT presenting ≥10 years after 1st TGCT [77]. Testicular ultrasound should be considered in years 3 and 5 in the presence of a normal contralateral testis, or more frequently in patients with risk factors or previous abnormal ultrasound findings such as microcalcifications.

On the other hand, new strategies are being developed to reduce the risk of cumulative radiation exposure. In this sense, replacing CT with MRI, using low-dose non-contrast CT and avoiding chest x-rays may be safe, at least for low-risk patients [78].

As poor adherence to post-treatment follow-up protocols can be associated with higher rates of relapse, delay in definitive therapy and unnecessary morbidity, a number of strategies are being developed to improve adherence, such as reducing the number of hospital visits and tests, or incorporating new technologies such as mobile health (m-health) [79].

Post 5-year follow-up lacks consensus and requires individual patient assessment. For chemotherapy-treated patients, the emphasis transitions from detecting tumor recurrence to managing late treatment effects and promoting overall health. Patients should be motivated to lead a healthy lifestyle to mitigate the risk of severe late effects such as secondary cancers and cardiovascular disease.

Finally, it is expected that in the near future, the incorporation of new biomarkers predictive of residual disease or relapse (e.g., miR-371a-3p) will allow better prediction of the risk of recurrence and facilitate follow-up, reducing costs, and exposure to ionizing radiation [80–82].

## Late toxicity and complications in long-term survivors

Although 95% of patients with TGCT are cured, survivors face potential late adverse effects and reduced quality of life. The frequency and severity of specific adverse events have been combined into a cumulative burden of morbidity (CBM) score for patients who had received cisplatin-based chemotherapy. At a median follow-up of 4.2 years 20% had a high/severe CBM score, and only 5% had no adverse health outcomes. Therefore, understanding the risk of long-term effects of therapy is important to optimize care in this population [83].

Secondary neoplasms, infertility, cardiovascular toxicity, metabolic syndrome, specific sequelae of chemotherapy including neurotoxicity, ototoxicity, pulmonary and renal toxicity, and psychosocial distress associating anxiety and sexual dysfunction are the major long-term toxicities in this population [84].

The relative risk of a second non-germ cell solid tumor is approximately doubled after radiotherapy or chemotherapy and usually occurs more than 10 years after treatment. The most common associated solid tumors are of gastrointestinal, urinary tract and soft tissue origin. The estimated cumulative risk of leukemia is 0.5 and 2% after cumulative etoposide doses of <2 and >2 g/m^2^, respectively, and occurs within 10 years of treatment. The relative risk of a second solid non-germ-cell tumor is approximately doubled after radiotherapy or chemotherapy and usually occur more than 10 years after treatment. The most frequently related solid tumors are of gastrointestinal, urinary tract and soft tissue origin. The estimated cumulative risk of leukemia is 0.5 and 2% after cumulative etoposide doses of <2 and >2 g/m^2^, respectively and emerge within 10 years after treatment [85].

Metabolic syndrome affects 8–32% of long-term TGCT survivors, who have almost double the risk compared to controls. Male hypogonadism is observed in 11–35% of this population. Several studies have shown an association between metabolic syndrome and chemotherapy and low testosterone levels in TGCT survivors [86, 87]. Patients should be counseled on healthy lifestyle, smoking cessation, physical activity and monitoring of blood pressure, cholesterol and testosterone levels during follow-up.

Chemotherapy-induced cardiovascular toxicity is the result of direct endothelial damage induced by cisplatin and indirect hormonal and metabolic changes [88]. Compared with the general population, patients with TGCT who received chemotherapy had a significantly higher relative risk of cardiovascular disease, ranging from 1.4 to 7.1. The incidence of angina, myocardial infarction or sudden cardiac death was 7%. Increased cardiovascular mortality (both from heart disease and cerebrovascular disease) was not associated with TGCT but with cisplatin-based chemotherapy, especially during treatment and at 10 years [89].

Pre-existing fertility problems can be exacerbated by chemotherapy, extended field radiotherapy and RPLND and are further reduced by treatment with combined modalities with high doses of cisplatin (>850 mg). Population-based studies in TGCT survivors have shown a slightly reduced overall fertility and more frequent use of assisted reproductive technology with a success rate of 50%. No increased risk of malformations has been found in children of TGCT survivors [90].

Long-term cisplatin-induced peripheral neuropathy was seen in 20–30% of patients 5–10 years after treatment and was associated with cumulative cisplatin dose, age, smoking and alcoholism. Symptomatic ototoxicity is also common, including tinnitus (59%), hearing loss (18%) or both (23%). Half of patients who received a cumulative cisplatin dose >400 mg/m^2^ reported tinnitus and hearing loss.

Finally, other toxicities, generally dose related, are more common in TGCT survivors than in the general male population. These include some degree of renal impairment (up to 30%), pulmonary fibrosis (5–10% of patients treated with bleomycin, which can be fatal in 1%), chronic fatigue (17%), anxiety disorders (17–38%), clinically significant depression (5–12%) [91].

Oncologists should be aware of all these possible complications that may occur in long-term survivors to counsel patients with preventive measures and, if necessary, to provide early diagnosis and treatment.

## Ethics statement

The current study has been performed in accordance with the ethical standards laid down in the 1964 Declaration of Helsinki and its later amendments.
